# Visualizing the Structure‐Property Nexus of Wide‐Bandgap Perovskite Solar Cells under Thermal Stress

**DOI:** 10.1002/advs.202401955

**Published:** 2024-05-29

**Authors:** Degong Ding, Yuxin Yao, Pengjie Hang, Chenxia Kan, Xiang Lv, Xiaoming Ma, Biao Li, Chuanhong Jin, Deren Yang, Xuegong Yu

**Affiliations:** ^1^ State Key Laboratory of Silicon and Advanced Semiconductor Materials School of Materials Science & Engineering Zhejiang University Hangzhou Zhejiang 310027 China; ^2^ Department of Chemistry Zhejiang University Hangzhou 310058 China; ^3^ Zhejiang University‐Hangzhou Global Scientific and Technological Innovation Center Hangzhou 310014 P. R. China

**Keywords:** in situ TEM, perovskite/silicon tandem, structure‐property nexus, thermal degradation mechanisms, wide‐bandgap perovskite

## Abstract

Wide‐bandgap perovskite solar cells (PSCs) toward tandem photovoltaic applications are confronted with the challenge of device thermal stability, which motivates to figure out a thorough cognition of wide‐bandgap PSCs under thermal stress, using in situ atomic‐resolved transmission electron microscopy (TEM) tools combing with photovoltaic performance characterizations of these devices. The in situ dynamic process of morphology‐dependent defects formation at initial thermal stage and their proliferations in perovskites as the temperature increased are captured. Meanwhile, considerable iodine enables to diffuse into the hole‐transport‐layer along the damaged perovskite surface, which significantly degrade device performance and stability. With more intense thermal treatment, atomistic phase transition reveals the perovskite transform to PbI_2_ along the topo‐coherent interface of PbI_2_/perovskite. In conjunction with density functional theory calculations, a mutual inducement mechanism of perovskite surface damage and iodide diffusion is proposed to account for the structure‐property nexus of wide‐bandgap PSCs under thermal stress. The entire interpretation also guided to develop a thermal‐stable monolithic perovskite/silicon tandem solar cell.

## Graphical abstract

The dynamic degradation process of wide bandgap perovskites solar cells (PSCs) induced by thermal stress under practical operating conditions is captured by in situ transmission electron microscopy, which is promising to provide significant guidance for seeking effective strategies for realizing the long‐term stability of PSCs.

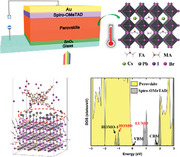

## 1 Introduction

Wide‐bandgap (WBG) metal halide perovskites are promising portions for monolithic tandem photovoltaics based on silicon, perovskite or organic sub‐solar cells, whose efficiencies surpass the limit of state‐of‐the‐art single junction cell considering effective light management with low non radiative recombination losses.^[^ ^1^, ^2^, ^3^ ^]^ Nevertheless, the long‐term stability is still of great challenge for tandem solar cells toward commercialization,^[^ ^4^, ^5^, ^6^, ^7^ ^]^ where the instability of WBG perovskite cells is a common and serious crux plagued by external conditions including moisture/oxygen, light, heat and etc.^[^ ^8^, ^9^, ^10^ ^]^ Amongst them, thermal‐input is a general existence in devices even though under normal working conditions of illumination and bias operations, which will result in significant structure and performance degradation in two parts. The first is the intrinsic instability of perovskite itself under thermal conditions, due to the easily broken lead‐halogen bonds and volatile organic cations.^[^ ^11^ ^]^ In addition, the heat‐induced interfacial deterioration in perovskite solar cells (PSCs) also tends to cause irreversible degradation of performance, e.g. interfacial diffusion behaviors of perovskite element migration into charge transport materials.^[^ ^12^, ^13^ ^]^ Hence, accurately observation of the heat‐driven device degradation is the prerequisite to fully understand degradation mechanisms and further seek effective approaches to address stability issues.^[^ ^14^, ^15^ ^]^

Protocols based on advanced transmission electron microscopy (TEM) are powerful tools for investigating the transformations of micro‐structures and chemical compositions,^[^ ^16^ ^]^ which can be devoted to unravel the thermal‐degradation mechanisms involved in the evolution of each function layer in WBG PSCs.^[^ ^17^, ^18^, ^19^, ^20^, ^21^, ^22^, ^23^, ^24^, ^25^, ^26^ ^]^ For example, high‐resolution TEM characterization revealed that tetragonal MAPbI_3_ experience an transition to PbI_2_ grains^[^ ^27^ ^]^ on heating, and PbI_2_ grains precipitated from MAPbI_3_ layer^[^ ^28^ ^]^ was also along a fixed crystallographic direction.^[^ ^29^ ^]^ However, most of the existing reports focused on the intrinsic stability of MAPbI_3_ under thermal stress and revealed the poor thermal tolerance of 2,2′7,7′‐tetrakis‐(N, N’, di‐p‐methoxyphenylamine)−9,9′‐Spirobifluorene (Spiro‐OMeTAD)^[^ ^30^, ^31^ ^]^ in MAPbI_3_ based PSCs,^[^ ^28^, ^32^ ^]^ while partially applied temperature values were divorced from reality.^[^ ^30^, ^33^, ^34^ ^]^ The entire structure‐property nexus of WBG‐PSCs under thermal stress is not yet thoroughly explored at microscopic‐scale, especially for the poly[bis(4‐phenyl)(2,4,6‐trimethylphenyl)amine] (PTAA)‐based PSCs. Because PTAA is one of the promising hole transport layer (HTL) candidates in PSCs due to its ease of fabrication, doping modification, transparency to visible light, mechanical flexibility, conductivity and stability, which is also utilized as the HTL in n–i–p and p–i–n PSCs with extended applications in flexible, large‐area, and tandem devices.^[^ ^35^ ^]^

In this study, nip PSCs with specific perovskite composition and different HTLs (Spiro‐OMeTAD or PTAA) were carried out via in situ heating inside TEM to capture the entire thermal degradation process in real‐time including structure transformations and elemental analysis, combining with the photovoltaic performances characterization. In the early stage at 50 °C, there is a morphology‐dependent degradation phenomenon that a spot of defects prefers to form at perovskite grain boundaries (GBs). As the temperature increased to 85 °C, defects at the perovskite surface aggravate more severely and detrimentally, iodine is found to diffuse into Spiro‐OMeTAD instead of PTAA with heat amplifying, leading to the Spiro‐OMeTAD‐based PSCs lost their performances dramatically but PTAA‐based PSCs sustained. Under more intense thermal conditions, produced PbI_2_ tends to appear at GBs and edges of the perovskite crystalline along with topo‐coherent interface of PbI_2_/perovskite. Based on the experimental results, we proposed a mutual inducement mechanism of perovskite surface damage and iodide diffusion and further confirmed by density functional theory calculations to reveal the structure‐property nexus of wide‐bandgap PSCs under thermal stress. These results also guided us to develop a PTAA‐based monolithic perovskite/silicon tandem solar cell which demonstrated competitive thermal stability.

## 2 Results and Discussion

### 2.1 Device Structure and Photovoltaic Performance Characterization

**Figure 1:**
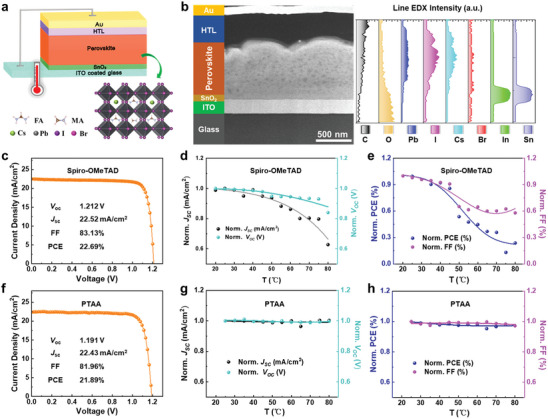
Planar PSCs device structure and photovoltaic performance under thermal operating: *V*_OC_, *J*_SC_, FF, and PCE. a) Schematic of the full device structure of planar PSCs. b) Cross‐sectional STEM image and corresponding line EDX intensity of the relevant elements in perovskite. c–e) *J–V* curve and performance parameters (*V*_OC_, *J*_SC_, FF, and PCE) of Spiro‐OMeTAD‐based PSCs under stepwise thermal operating. The photovoltaic performance values are normalized by the initial values obtained at room temperature for the cell. f–h) *J–V* curve and corresponding parameters of PTAA‐based PSCs performance under same operated conditions with Spiro‐OMeTAD‐based PSCs.

The full device structure of indium tin oxide (ITO)/SnO_2_/perovskite/HTL(Spiro‐OMeTAD or PTAA)/Au developed here is schematically illustrated in **Figure** **1**a, of which light absorption layer is mixed cation organic–inorganic lead halide perovskites Cs_0.05_FA_0.8_MA_0.15_Pb(I_0.75_Br_0.25_)_3_. The corresponding structure characterization of the PSCs with Spiro‐OMeTAD as an example is presented in Figure 1b and Figure S1 (Supporting Information), by a representative cross‐sectional annular dark‐field scanning transmission electron microscopy (ADF‐STEM) image and energy‐dispersive X‐ray spectroscopy (EDX) maps, displaying a ≈700 nm thick perovskite film with homogeneous composition distributions. The bandgap of the perovskite absorber is determined to be 1.68 eV by UV–vis and photoluminescence spectroscopies (Figure S2, Supporting Information).

The photovoltaic performances of Spiro‐OMeTAD and PTAA‐based PSCs are reported in Figure 1c–h. The typical *J–V* curves of opaque (Figure 1c,f) and semi‐transparent single‐junction PSCs (Figure S3, Supporting Information) show the initially high efficiencies for the two PSCs. The temperature‐related photovoltaic properties characterization of the Spiro‐OMeTAD‐based PSCs illustrated a significant decrease in the *V*_OC_ and *J*_SC_ (Figure 1d) as temperature increased, causing decrease in the overall performance of PCE and FF (Figure 1e). For the thermal operation at 80 °C, almost all photovoltaic parameters are deteriorated, where the PCE only retain 26% of the initial value. On the contrary, the PTAA‐based PSC is much more stable, where the PCE still remains above 95% of the initial value even at 100 °C (Figure 1h), only with little decayed of *V*_OC_, *J*_SC_ (Figure 1g, within 0.5% loss) and FF. Specifically, we note that performance deterioration is relatively more moderate within 50 °C in the early period while becomes sharper later with almost 75% PCE loss at 80 °C for the Spiro‐OMeTAD‐based PSC, indicating the existences of different structural destruction mechanisms in the device at various temperatures. Following, we carry out in situ TEM to visualize the thermal degradation process of the complete devices configuration corresponding to their performance evolutions under the thermal stress.

### 2.2 Morphology‐Dependent Microstructure and Composition Evolution of Perovskite Under Thermal Operation

**Figure 2:**
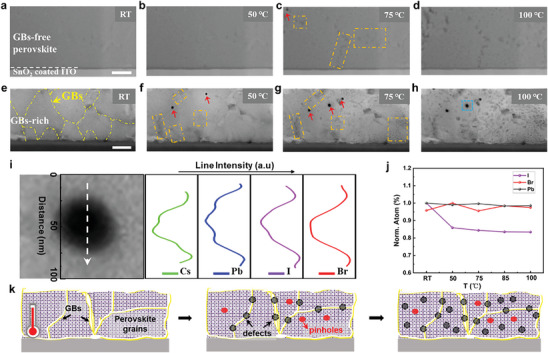
Morphology‐dependent thermal degradation and component analysis of planar perovskites. a–d) Surface defects formation of GBs‐free and e–h) GBs‐rich perovskite film under different thermal condition from room temperature to 100 °C. Scale bar: 200 nm. i) The line scanning EDX analysis of the pin‐holes showed the main element loss in perovskite. Left panel: STEM image of pin‐hole region, white arrow is line scanning direction. Right panel: corresponding line profile intensity of elements in perovskite and the concave peak shape toward the left means the element intensity is reduced. j) The content changes of different elements in perovskite from quantitative analysis of EDX mapping. k) Schematic illustration of thermal degradation of GBs‐rich perovskite film.

**Figure** **2** illustrates the microstructural and composition evolution of the perovskite film deposited on the SnO_2_ coated ITO substrate with two different morphologies under different temperatures in cross‐sectional views, respectively. The cross‐sectional ADF‐STEM images in Figure 2a–d shows the perovskite film with GBs‐free morphology, indicating no appearance of surface defects in perovskite within 50 °C (Figure 2a,b), and slight deficit of compositions occurs until the operating temperature increase to 75 °C (marked with orange dashed rectangle in Figure 2c). The defects become aggravated at high operating temperature of 100 °C (Figure 2d). However, the morphological evolution for the GBs‐rich perovskite film is different, as shown in Figure 2e–h and Figure S4 (Supporting Information). Apparent defects are prone to appear at GBs (marked by orange dash rectangle) and apparent pin‐holes (denoted by red arrows) formed at the moderate temperature of 50 °C (Figure 2f). At higher temperatures of 75 °C and 100 °C, the degradation of GBs‐rich perovskite is much more serious than that of GBs‐free perovskite, as presented in Figure 2g,h. Further line scanning EDX analysis of pin‐holes region indicates the main element deletion (Cs, Pb, I, Br) in perovskites, as shown in Figure 2i and Figure S5 (Supporting Information). The surface morphology characterization conducted by SEM (Figure S5b, Supporting Information) also show that no changes of pinholes are observed as the temperature increased from 50 °C to 100 °C. These pinholes may be attributed to the volatilization of the residual organic solvents,^[^ ^36^ ^]^ and they are verified to occur in both perovskite films (marked with red arrows in Figure 2c,f) and be irrelevant to the further rise of the operating temperature (Figure 2h). Moreover, quantitative analysis of elements contents (detailed in the Experimental Section) was carried out to demonstrate the major component fluctuations in perovskite at different thermal stages. The normalized elements content in perovskite matrix presented in Figure 2j indicates the obvious iodide deficiency compared to Pb and Br elements sustained in a stable range with the stepwise increased temperature. In view of the above results, we deduce that these defects driven by heat might be due to the release of the thermal unstable MA accompanied with iodide deficiency,^[^ ^29^ ^]^ which preferred to turn out at GBs although at moderate thermal conditions, as depicted in schematic diagram of Figure 2k.

### 2.3 Perovskite Surface Damage and Iodide Diffusion

**Figure 3:**
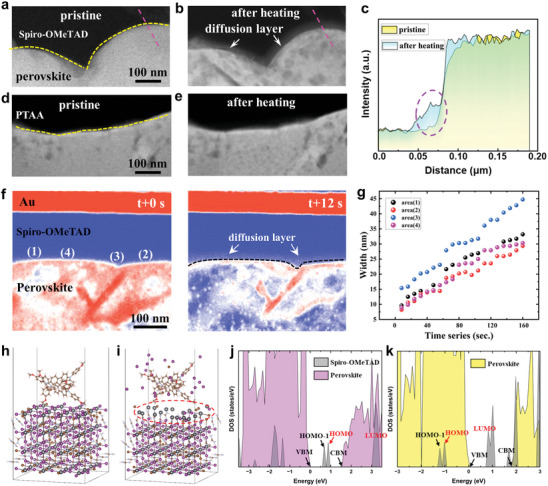
Iodide ions diffusion at the interface of perovskite/HTL and effects on HTL electronic structures. a,b) ADF‐STEM images showing cross‐sectional views of the perovskite/Spiro‐OMeTAD and d,e) perovskite/PTAA interface before and after heating. c) The line profile showing the intensity difference between pristine and heating at perovskite/HTL interface. f) Time sequential ADF‐STEM images with false‐color of the diffusion region expanding at elevated temperature of 200 °C. g) The corresponding statistical analysis of the thickness of four different regions in f. h,i) Side view of fully relaxed atomic models of perovskite slabs and HTL (Spiro‐OMeTAD as an example). j) Density of states of the pristine Spiro‐OMeTAD molecule on perfect perovskite surface. k) Density of states of the iodide ions diffused Spiro‐OMeTAD molecule on perovskite surface, iodide ion concentration is 16%.

The behavior of ions diffusion is analyzed further by in situ ADF‐STEM with Z‐contrast imaging mechanism and EDX in **Figures** **3** and S6 (Supporting Information). Iodide diffusion into Spiro‐OMeTAD has been monitored because of the high mobility of the iodide ions in previous reports,^[^ ^30^, ^32^, ^37^ ^]^ even at relatively low temperature <85 °C,^[^ ^31^ ^]^ which is also consistent with our results in Figure 3a–c. The material basis of the diffusion layer is iodide‐doped Spiro‐OMeTAD, which is verified by EDS, HRTEM, and theory calculation in details there in after. However, iodine diffusion in PTAA‐based PSCs is distinctive, which no apparent diffusion layer observed at the interface of PTAA/perovskite at 85 °C, as shown in Figure 3c–d. For more details, the HRTEM and FFT are conducted to characterize the interface of perovskite/HTL (Figure S7, Supporting Information). The diffusion region proximal to perovskite top surface is amorphous reflected by the FFT image, indicating an ion diffusion occurrence without any specific crystallize formation in Spiro‐OMeTAD. In addition to iodide diffusion, there are severer damage in Spiro‐OMeTAD based perovskite matrix (Figure 3b) than that of the PTAA‐based perovskite matrix (Figure 3e). The dynamics of the expansion of diffusion region are illustrated by time‐sequenced ADF‐STEM images (Movie S1, Supporting Information). As seen in Figure 3f at t+0 s, the false‐color ADF‐STEM image shows the clear interface structure between top Spiro‐OMeTAD and bottom perovskites, no extra contrast is observed at the interface compared to the diffusion region shown in Figure 3b. While as time goes on, a significant contrast can be observed in Spiro‐OMeTAD approaching to perovskite surface and constantly increasing to approximately ten of nanometers at t+12 s (Figure 3g). It is also noted that the contrast of terminated‐surface (perovskite top‐surface approach to Spiro‐OMeTAD layer) marked by black dash rectangle in Figure S8 (Supporting Information) and perovskite matrix is descending gradually in the meantime. We can deduce that the terminated‐surface and matrix of perovskite is damaged under continuous thermal treatment and iodide missed according to the descending contrast in Figure 3g and quantitative EDX results in Figure 2j.

We carried out density functional theory (DFT) calculations to capture the fully relaxed interface structure of perovskite/HTL before and after iodide diffusion, taking Spiro‐OMeTAD as an example. Since the interaction between Spiro‐OMeTAD and perovskite is weakly physisorption, the atomic models were established by laying a Spiro‐OMeTAD monolayer on top of perovskite slabs with Pb─I termination, with the optimized interfacial structure as shown in Figure 3h. We compared the iodide concentration in Spiro‐OMeTAD of ≈16% in Figure 3i with that of ≈8% in Figure S9 (Supporting Information). It is exhibited that perovskite terminated‐surface appear to damage after sufficient structural relaxation when iodide exist in incompact Spiro‐OMeTAD, and the damage is severer along with higher concentration of iodide diffusion (marked by red circle in Figure 3i; Figure S9a, Supporting Information). These are consistent with the experimental results in Figure 3f. We further unraveled the electronic structure of the hybrid interface between Spiro‐OMeTAD and perovskite, which can be illustrated by the projected density of states (PDOS) after charge redistribution,^[^ ^38^ ^]^ as shown in Figure 3j,k and Figure S9b (Supporting Information). Compared with the pristine interfacial configuration in the absence of iodine diffusion (Figure 3j), the degenerated highest occupied molecular orbital (HOMO) and HOMO‐1 (degenerated energy levels) of Spiro‐OMeTAD are deepened and exceed the valence band maximum (VBM) of perovskite due to the diffusion of iodide, which will form an interface barrier for obstacle hole transferring to the Spiro‐OMeTAD layer, thereby greatly decreasing the efficiency of charge separation^[^ ^39^ ^]^ across the interface (Figure 3k). Combining with the experimental results in Figure 3a–g, a mutual inducement mechanism can be determined to account for thermal degradation and iodide diffusion. The damaged terminated‐surface leaves channel‐like path since then a continuous iodide flow into the HTL layer under thermal conditions, which is similar to the layer‐by‐layer degradation procedure,^[^ ^29^ ^]^ and form diffusion regions. The schematic illustration of the whole dynamic process is depicted in Figure S9c (Supporting Information) and also described in detail below.

From the above results, we can now propose intuitive complementary aspects explaining the structure‐property nexus between PSCs thermal degradation and photovoltaic performance loss. Even though the moderate heat will generate the partial composition deficiency inside the bulk perovskite, it degrades the device performance not as seriously as expected might due to the resultant shallow level defects via organic cation or iodide vacancies in a short‐term operation period.^[^ ^40^, ^41^ ^]^ By comparison, the perovskite surface appears to be more sensitive to the device performance since it is commonly more defective as a crystal growth cutoff, leading to preferential structure damage and component transformation. Here, it is deduced that the perovskite surface damage induced by thermal stress leaves channel‐like path in perovskite matrix for iodide diffusion into Spiro‐OMeTAD, leading to the interface energy level mismatch and then descending the photovoltaic performance. We can exclude the intrinsic structural destruction of Spiro‐OMeTAD since the glass transition temperature of Spiro‐OMeTAD itself should be 125 °C.^[^ ^42^, ^43^ ^]^ The dopants including Li‐salt and tBp concerning to the thermal stability require in‐depth exploration later. Whilst, the PSCs with dense and compact PTAA HTLs can demonstrate better thermal stability on account of neglect iodide diffusion into PTAA <100 °C, according to our experimental results. Hence, it is essential to functionalize Spiro‐OMeTAD layer by molecule engineering^[^ ^44^, ^45^ ^]^ or build up a dense barrier‐layer at perovskite/Spiro‐OMeTAD interface,^[^ ^9^ ^]^ such as metal oxides^[^ ^46^ ^]^ and organic self‐assembled monolayers,^[^ ^47^, ^48^ ^]^ hindering ions diffusion crossing the interface without comprising the conductivity of Spiro‐OMeTAD. Upon the interface engineering, for a long‐term device stability consideration, strengthening the stability of perovskite lattice itself, such as designing MA‐free WBG perovskites^[^ ^49^, ^50^, ^51^ ^]^ or diminishing and passivating GBs,^[^ ^52^ ^]^ is a conspicuous direction in the future for real industrialization photovoltaics applications with WBG perovskites.

### 2.4 In Situ TEM Observation of a General Decomposition Pathway of Planar Perovskites

**Figure 4:**
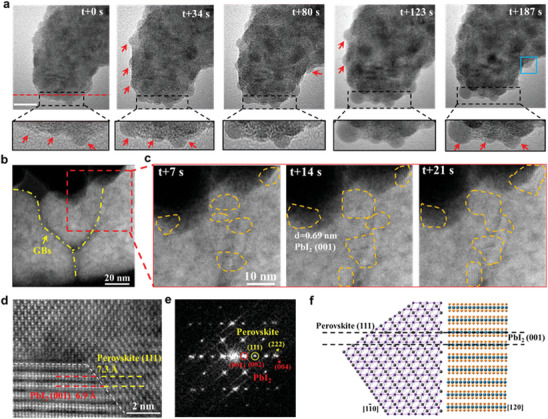
In situ TEM investigation the decomposition pathway. a) Time‐sequenced HRTEM images of perovskite grain evolution under continuous electron beam illumination. The edge regions are zoomed‐in rectangle framework images. Scale bar: 10 nm. b) ADF‐STEM image of planar perovskite grain with nearly triple‐symmetric grain boundary. c) Time‐lapsed ADF‐STEM images of PbI_2_ formation and growth at grain boundary and edge of perovskite. d) Atomic‐resolved ADF‐STEM image of topo‐coherent interface of perovskite/PbI_2_, and e) its corresponding FFT image and f) atomic models.

Additionally, to further elucidate the microstructure heterogeneity and decomposition pathway of the planar perovskite at more intense thermal conditions, we conducted in situ high‐resolution (HR)‐TEM to capture the dynamics of phase transition using electron beam as thermal source for real‐time observation. This approach has also been proved to be effective to study the dynamics behaviors induced by thermal stress of both in perovskites^[^ ^22^, ^27^, ^34^, ^53^, ^54^ ^]^ and other crystal systems.^[^ ^55^, ^56^ ^]^ The time‐sequential HRTEM image series represent the detailed transformation dynamics of perovskite grains under continuous e‐beam illumination (**Figure** **4**a). At t+0 s, the perovskite grain is uniform, and apparent nanoparticles precipitate at the edges (as signed with red arrows) of perovskite crystalline at t+34 s, and these nanoparticles increase and grow up gradually until t+187 s. The enlarged images in the black dotted frame better illustrates this dynamic process. The crystallization information of the edge nanoparticles is clarified by atomic‐resolved ADF‐STEM images in Figure S10 (Supporting Information), presenting monoclinic crystallographic form, together with associated spacing of 0.261 nm for the (102) plane of PbI_2_ family.^[^ ^28^ ^]^

We selected another time‐lapsed ADF‐STEM image of the planar perovskite grains with triangle GBs shown in Figure 4b,c, directly illustrating that PbI_2_ as the decomposition product is prone to segregate at the GBs or the edges of the perovskite under constant thermal energy transferred from electron beam, which is consistent with the results shown in Figure 4a. The interface cutoffs are generally defective and act as ion migration and defect aggregation channels, resulting in the origin of structural or composition transformations. Then we took a deep look into the interface of PbI_2_/perovskite in Figure 4d and revealed the dependence crystal structure of perovskite to PbI_2_. It identifies the topo‐coherent crystal planes of PbI_2_/perovskite with (111)_perovskite_ // (001)_PbI2_ (Figure 4d), along with the representative crystal axis on perovskite [$1\bar{1}$0] and PbI_2_ [120]. And the fast Frontier transformation (FFT) image shows a good fit with simulated diffraction patterns, wherein (111)_perovskite_ and (001)_PbI2_ exactly exhibit an almost identical hexagonal close‐packing pattern (Figure 4e; Figure S11, Supporting Information), which can be regarded as the reversed process of topochemical assembly mechanism of PbI_2_/FAPbI_3_.^[^ ^57^ ^]^ Based on the above analysis, we suppose the solid–solid transition mechanism in which perovskite thermal degraded to PbI_2_ may follow an alternative pathway along the crystallographic orientation at coherent interface of PbI_2_/perovskite, as schematic illustrated in Figure 4f.

### 2.5 Thermal‐Stable Monolithic Perovskite/Silicon Tandem Solar Cells

**Figure 5:**
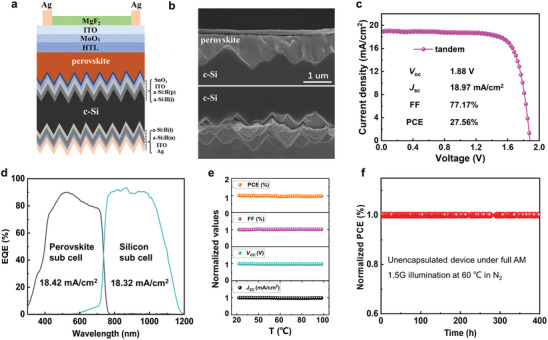
PTAA‐based Perovskite/ silicon tandem solar cells. a) Cross‐sectional sketch of the perovskite/c‐Si bifacial tandem device and b) corresponding SEM image of the tandem realized on a both‐sides textured c‐Si bottom cell. c) Representative *J–V* curve and performance parameters (PCE, *V*_oc_, FF, and *J*_sc_) of PTAA‐based tandem PSCs. d) EQE curves of the tandem PSCs. e) Performance parameters under stepwise thermal operating. The photovoltaic performance values are normalized by the initial values obtained at room temperature for the cell. f) MPP tracking stability tests of the tandem PSCs.

Currently and prospectively, monolithic perovskite/silicon tandem solar cells are a living benchmark,^[^ ^58^, ^59^ ^]^ calling for of quality WBG perovskites, for achieving high efficiency and stability. According to our experimental explorations, the PTAA‐based single‐junction PSCs exhibit considerable thermal‐stability, which is promising for constructing efficient and thermal‐stable perovskite/silicon tandem solar cells. As a consequence, we developed the fully textured nip monolithic perovskite/silicon tandem solar cells using bifacial textured SHJ as a bottom cell as schematically sketched in **Figure** **5**a. This yields the perovskite film fully covering the micrometer‐sized Si pyramids, as confirmed by the SEM image shown in Figure 5b. The representative *J–V* characteristics of the PTAA‐based tandem devices are shown in Figure 5c, featuring a PCE of 27.56% with a *V*_OC_ of 1.88 V, a *J*_SC_ of 18.97 mA cm^−2^ and an *FF* of 77.17%, which is more superior than that of Spiro‐OMeTAD based tandem device (Figure S12, Supporting Information). The integrated *J*_SC_ value of the perovskite is 18.42 mA cm^−2^, which agrees well with that of silicon sub cell of 18.32 mA cm^−2^ (Figure 5d). The temperature‐related photovoltaic performance of the tandem PSCs is carried out under stepwise thermal operating from room temperature to 110 °C. No significant decrease of all photovoltaic parameters is observed as temperature increases (illustrated in Figure 5e). Moreover, the MPP tracking test is performed under 1‐sun illumination for 400 h at 60 °C in N_2_, showing no efficiency attenuation and excellent stability of the PTAA‐based tandem devices (Figure 5f) compared with the Spiro‐OMeTAD‐based device (Figure S13, Supporting Information). These also confirms the great thermal‐tolerance potential of PTAA‐based perovskite/silicon tandem solar cells.

## 3 Conclusion

In summary, we revealed the thermal degradation process WBG PSCs with two different HTL layers of Spiro‐OMeTAD and PTAA respectively, in conjunction with in situ transmission electron microscopy (TEM) and corresponding to photovoltaic performance characterization. It is illustrated a morphology‐dependent degradation circumstances that defects are preferred to appear at grain boundaries at moderate heating stage (≈50 °C). Afterward, the decomposed product PbI_2_ occurred at the GBs and edges of perovskite grains preferentially along with a topo‐coherent interface of PbI_2_/perovskite at more intense thermal conditions. According to a mutual inducement mechanism, highly mobile iodine diffusing into HTLs along with perovskite surface damage under thermal stress significantly deteriorates device performance and stability. We further develop PTAA‐based perovskite‐silicon tandem solar cells, indicating well thermal‐stability under stepwise thermal operating with no apparent properties parameters degradation. These results provided an entire structure‐property nexus between perovskite degradation and device performance loss, providing exploratory insights for the high thermal‐stability and efficiency PSCs.

## 4 Experimental Section

### Materials

Lead iodide (PbI_2_, 99.999%), Lead bromine (PbBr_2_, >99%), Cesium iodide (CsI, 99.99%), Methylammonium chloride (MACl, 99.9%), Methylammonium bromide (MABr, 99.9%) and Poly(bis(4‐phenyl)(2,4,6‐trimethylphenyl)amine) (PTAA, >99%) were purchased from Xi'an Polymer Light Technology Corporation. Formamidinium iodide (FAI, 99.5%), Spiro‐OMeTAD (99.8%), and ITO were purchased from Advanced Election Technology Co., Ltd. Tin(IV) oxide (SnO_2_, 15% colloidal solution) was purchased from Alfa Aesar. 4‐Isopropyl‐4′‐methyldiphenyliodonium tetrakis (pertafluorophenyl) broate (TPFB) was purchased from Tokyo Chemical Industry Co., Ltd. All solvents and all other chemicals were purchased from Sigma–Aldrich and used as received.

### Fabrication of Single‐Junction Perovskite Solar Cells

The ITO‐coated glass substrates were sequentially cleaned with deionized water, acetone, ethanol, and isopropanol, followed by ultraviolet‐ozone for 20 min after drying in an N_2_ stream. Tin oxide (SnO_2_) nanocrystal aqueous solution diluted five times with deionized water was spin coated on UV‐cleaned ITO at 3000 rpm for 30 s, followed by annealing at 150 °C for 30 min. The SnO_2_ substrates were treated with UVO for 20 min before transferred into the glovebox to conduct the following process. The perovskite precursor solutions were prepared in a mixed solvent of DMF and DMSO (volume ratio 4:1). Perovskite films were prepared by the anti‐solvent method. The perovskite films were deposited onto the SnO_2_ substrates with a two‐step spin‐coating procedure. The first step was 2000 rpm for 10 s with an acceleration of 200 rpm s^−1^. The second step was 6000 rpm for 40 s with a ramp‐up of 2000 rpm s^−1^. Chlorobenzene (100 µL) was dropped on the spinning substrate during the second spin‐coating step at 20 s before the end of the procedure. The post‐annealing process was done at 100 °C for 30 min. Then the hole transport layer (HTL) solution, in which a Spiro‐OMeTAD/CB (72.25 mg mL^−1^) solution was employed with the addition of 17.5 µL Li‐TFSI/acetonit rile (520 mg mL^−1^), and 28.75 µL 4‐tertbutylpyridine, was deposited by spin‐coating at 3000 rpm for 30 s. In addition, PTAA HTL was prepared in chlorobenzene solution (30 mg mL^−1^), and doped with 11% TPFB. The doped PTAA solution was stirred at 45 °C overnight to ensure full dissolving and doping of PTAA. PTAA solution was spun on perovskite film at 4000 rpm for 30 s, followed by annealing at 80 °C for 5 min. Last, 100 nm of gold was evaporated onto the device with shadow mask to determine the device area.

### Fabrication of Perovskite/Silicon Tandem Solar Cells

The fabrication procedures of silicon bottom‐cell are the same as the previous studies.^[^ ^60^ ^]^ The difference was that it needed to be turned upside down and a smaller pyramid structure was prepared on the front side. For the fabrication of the perovskite top cells, the same SnO_2_ deposition as described above was implemented on the wafer. The Si bottom wafers were subjected to UV‐Ozone treatment for 10 min before transferred into the glovebox. 1.7 M Cs_0.05_FA_0.8_MA_0.15_Pb(I_0.75_Br_0.25_)_3_ perovskite precursor solution was prepared by dissolving a mixture of FAI, MABr, CsI, PbI_2_, and PbBr_2_ in a mixed solvent (DMF/DMSO  = 4:1). The perovskite films were spin‐coated at 2000 rpm for 50 s then followed with 7000 rpm for 10 s. Chlorobenzene was dropped in the center of the substrates 12 s before the end of the spin‐coating process. The films were immediately transferred onto a hotplate of 100 °C and were annealed for 15 min. A solution of PTAA/toluene (10 mg mL^−1^) with an additive of 7.5 µL Li‐bis(trifluoromethanesulphonyl) imide/acetonitrile (170 mg mL^−1^) and 4 µL 4‐tert‐butylpyridine was spin‐coated on the perovskite substrate at 3,000 rpm for 30 s. Then, 10 nm MoO_3_ was thermally evaporated on PTAA films. For IZO deposition, a 3‐inch IZO ceramic target containing 90 wt.% In_2_O_3_ and 10 wt.% ZnO was used. 30 sccm Ar with 0.5% O_2_ was used a process gas, yielding a ≈0.3 pa process pressure. 80 nm IZO was sputtered on top of the MoO_3_ through a shadow mask. Ag finger with a thickness of 400 nm was thermally evaporated using a high‐precision shadow mask. Finally, 120 nm MgF_2_ was thermally evaporated as an anti‐reflection layer.

### Device Performance Characterization

The photovoltaic parameters (*V*_OC_, *J*_SC_, FF, and PCE) of the PSCs were measured in a nitrogen (N_2_) filled glovebox under stepwise thermal operating from RT to 80 °C with every 5 °C, which was kept for 2 min until the temperature reaches the required before *J–V* measurements. Note that the temperature of the device was monitored using an infrared thermometer. The current density–voltage measurement (*J–V*) using an AM 1.5G simulator (94022A, Newport) in the air, calibrated by a standard Si solar cell (PVM937, Newport) with KG5 filter. *J–V* information was collected by Keithley 2400. Tandem devices were measured in forward scan (−0.2 V→2.0 V), and the scan rate was 10 mV s^−1^. External quantum efficiency (EQE) spectra of devices were tested using a QE system (Model QEX10, PV Measurements, Inc.).

The thermal stability of tandem devices was tested by recording the power conversion efficiency (PCE) every 12 h under one Sun‐equivalent white LED lamp in a N_2_ box. Simultaneous maximum‐power‐point (MPP) tracking of perovskite/silicon tandem solar cells was determined by a *J–V* sweep. Each cell was held at a specific voltage and the resulting current was measured in a N_2_ test box under one Sun‐equivalent white LED lamp. The devices were continuously heated at 80 °C.

### TEM Samples Preparation

The cross‐sectional perovskite solar cells (PSCs) samples were prepared using modified focus ions beam (FIB) technique in FEI Helios G4 system, including protecting layer deposition and sample thinning.^[^ ^61^ ^]^ The sample was first covered by amorphous carbon and Pt protection layer deposited by electron beam and ion beam in dual beam system. In order to reduce sample damage by ion beam, low‐voltage/current density thinning at 5 kV/70 pA and 1 kV/30 pA and relatively thick sample (≈150 nm) was conducted. And then a precision polishing at 2 kV was applied to reduce any surface damage and contaminations as soon as possible. Figure 2a,d illustrates a well‐maintained grain structure without apparent defects in its surface, indicating no damage was introduced during FIB process.

The planar perovskite samples were prepared by deposition the precursor suspensions onto holey carbon copper girds and then annealed at 120 °C for 10 min. The whole process was inside an argon‐filled glovebox to prohibit side reactions.

### Materials Characterization

The ADF/BF‐STEM and STEM‐EDX mapping characterizations were mainly conducted with a probe spherical aberration‐corrected scanning TEM (FEI‐Titan ChemiSTEM G2) operated at an acceleration voltage of 200 kV. The ADF‐STEM images were captured with low‐dose imaging process to diminish the electron beam irradiation effects. In situ heating STEM characterizations in Figures 2 and 3 were conducted by transferring the FIB lamella onto the heating chips (Wildfire S3 Nano‐chip, DENSsolution) loaded into a heating holder (DENSsolution, SH70). In situ, HRTEM characterization in Figure 4a was at FEI Tecnai F20 at 200 kV with a dose of 1900 e Å^−2^ to provide enough thermal energy and uniform irradiation.^[^ ^27^ ^]^ The elements quantitative analysis was made using EDX mapping in the STEM, which selected certain region in the EDX mapping image to get the elements quantitative values via the EDX software.

The top‐view SEM images of perovskite films were characterized by field‐emission Scanning Electron Microscopy (SU‐70, Hitachi)

Steady‐state PL spectra of the perovskite films were measured with a laser scanning confocal microscope (Witec RAS300) equipped with a 473 nm laser source.

The absorbance was performed using a UV–vis spectrometer (U‐4100, Hitachi Limited) employed with an integrating sphere.

### DFT Calculations

First principles energetic and electronic structure calculations were carried out within the framework of the density‐functional theory using projector augmented wave (PAW) pseudopotentials as implemented in the Vienna ab initio simulation package (VASP).^[^ ^62^, ^63^, ^64^ ^]^ The generalized gradient approximation (GGA) formulated used by Perdew, Burke, and Ernzerhof (PBE) as an exchange correlation functional.^[^ ^65^ ^]^ A kinetic energy cutoff of 400 eV for the plane wave basis set was generated. The Brillouin zone was sampled with Γ‐centered 1 × 1 × 1 k‐points. The energy and force convergence criteria were set to 10^−5^ eV and 0.01 eV Å^−1^.

## Conflict of Interest

The authors declare no conflict of interest.

## Supporting information

Supporting Information

Supplemental Movie 1

## Data Availability

The data that support the findings of this study are available from the corresponding author upon reasonable request.
