# Supplementary material for: Visualizing the Structure‐Property Nexus of Wide‐Bandgap Perovskite Solar Cells under Thermal Stress
Source: Adv Sci (Weinh). 2024 May 29;11(29):2401955. doi: 10.1002/advs.202401955 (PMC11304240; doi:10.1002/advs.202401955)
Supplement: Supplementary file 1 — Supporting Information [file ADVS-11-2401955-s002.docx]

**Supporting information**

**Visualizing the Structure-Property Nexus of Wide-Bandgap Perovskite Solar Cells under Thermal Stress**

*Degong Ding, Yuxin Yao, Pengjie Hang***, Chenxia Kan, Xiang Lv, Xiaoming Ma, Biao Li, Chuanhong Jin, Deren Yang, Xuegong Yu**


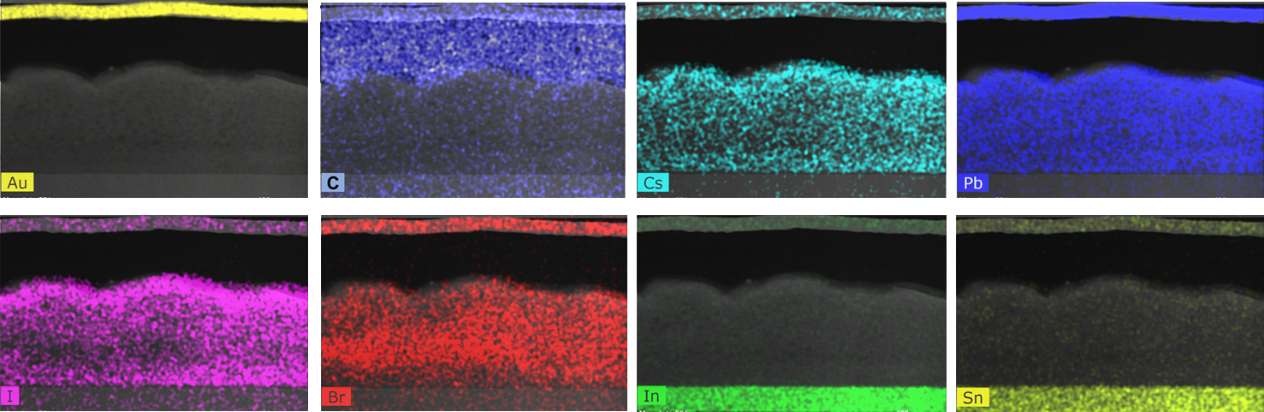


**Figure S1. EDX mapping.** The scanning transmission electron microscopy (STEM) energy-dispersive X-ray spectroscopy (EDX) maps of the perovskite solar cell (PSCs).


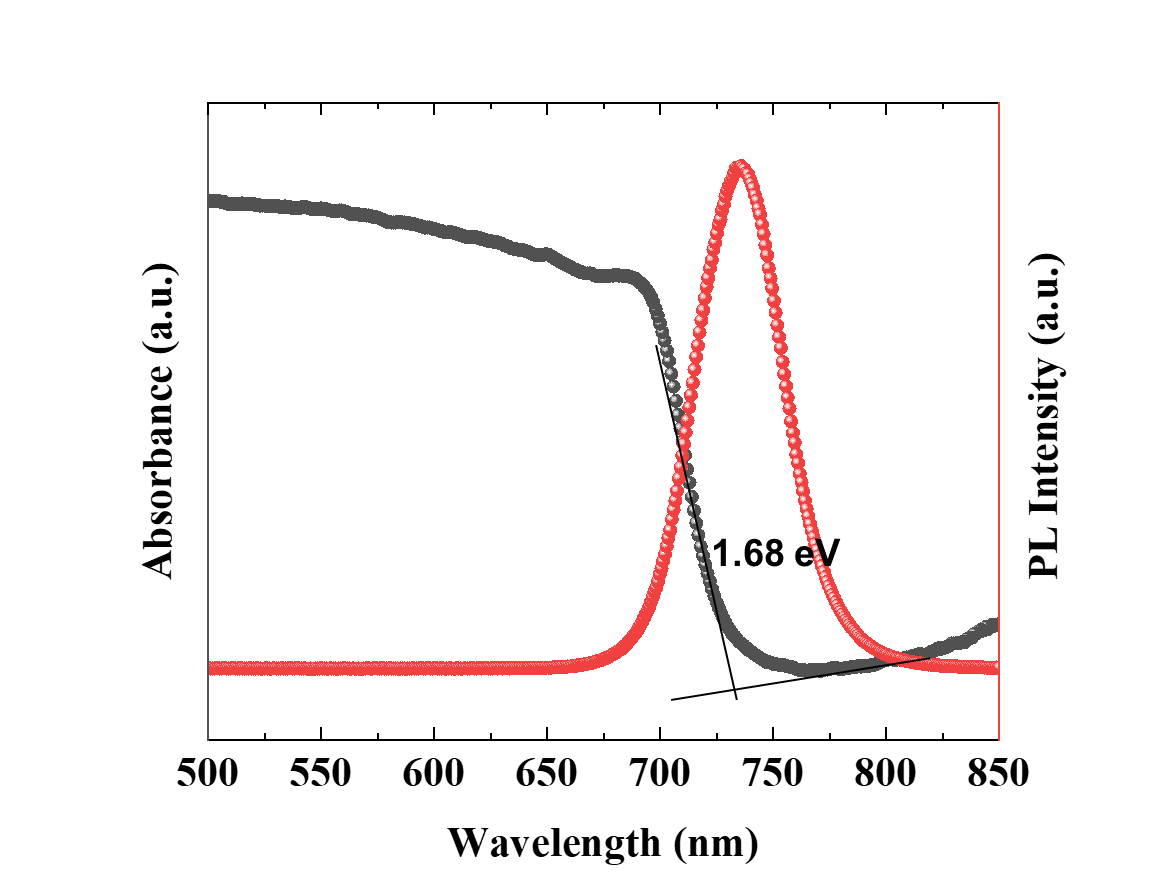


**Figure S2.** **Absorption and PL spectra.** Violet-visible (UV-vis) absorptions and photoluminescence (PL) spectroscopies of perovskite film.


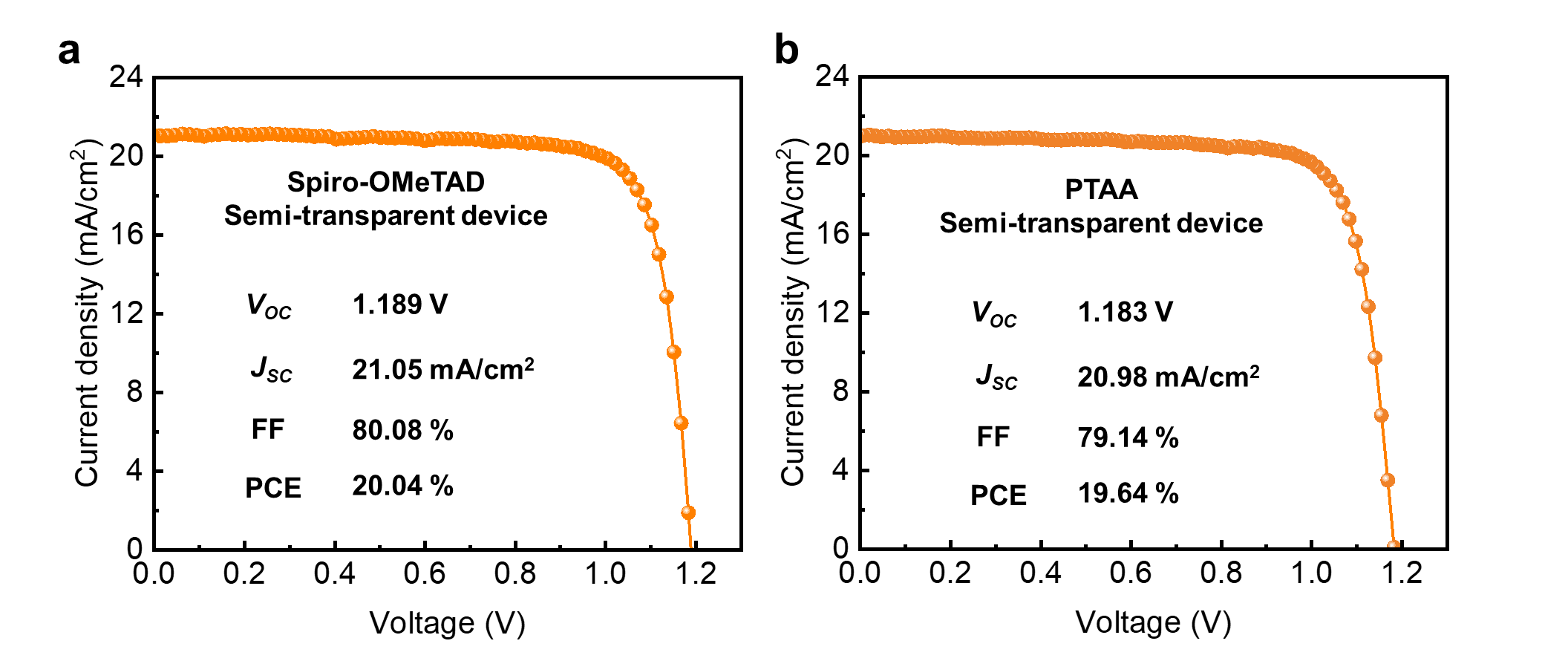


**Figure S3. The thermal stability tests.** *J-V* curves of semi-transparent device.


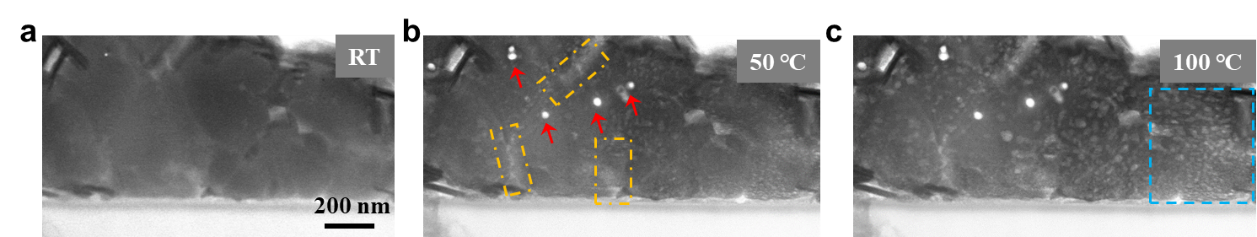


**Figure S4. Cross-sectional views under different thermal treatment.** Cross-sectional BF-STEM images of perovskite at different stages of heating.


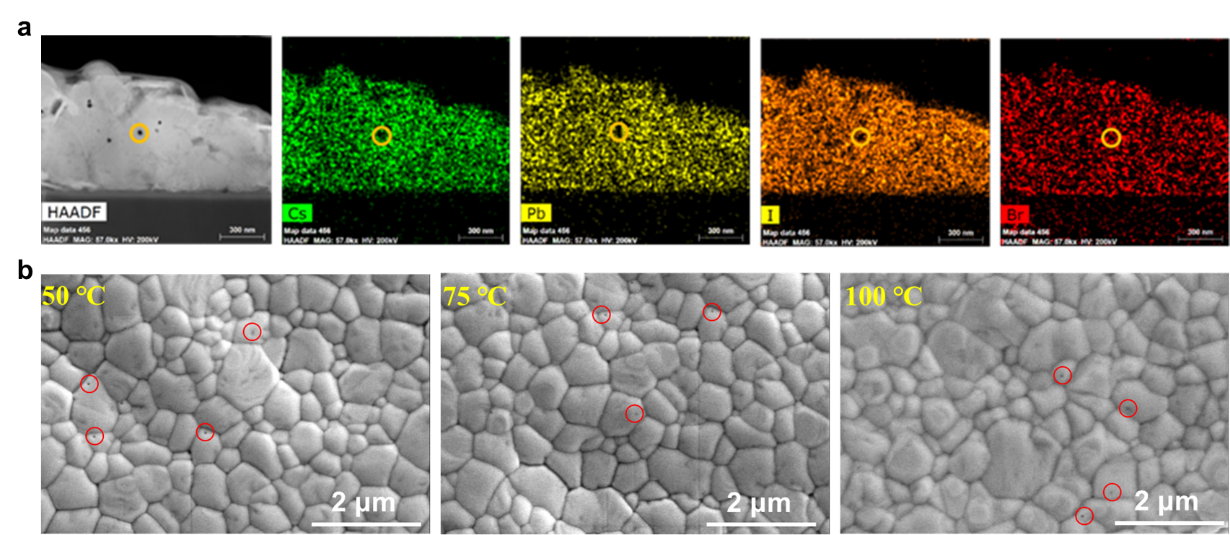


**Figure S5. Analysis of pin-holes.** **a**) EDX mappings of pin-holes region indicating the main element deletion (Cs, Pb, I, Br) in perovskite. **b)** SEM images of the perovskites surface morphology under different temperatures treatment from 50 ℃ to 100 ℃. The pinholes are marked by red circles.


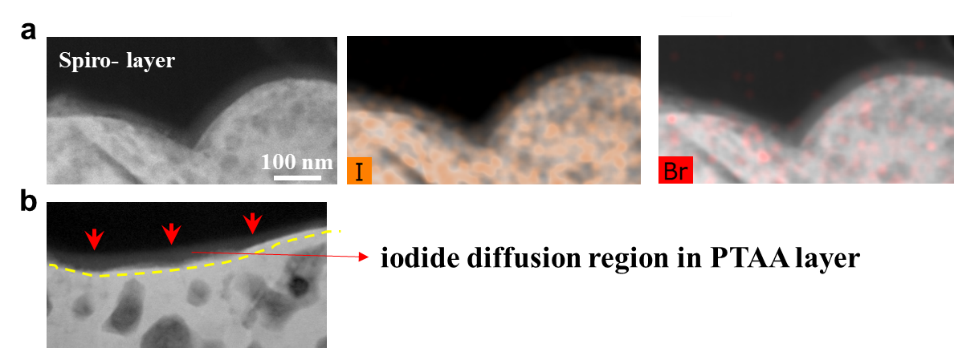


**Figure S6. Cross-sectional ADF-STEM images.** ADF-STEM images and EDX mapping showing cross-sectional views of the perovskite/Spiro-OMeTAD.


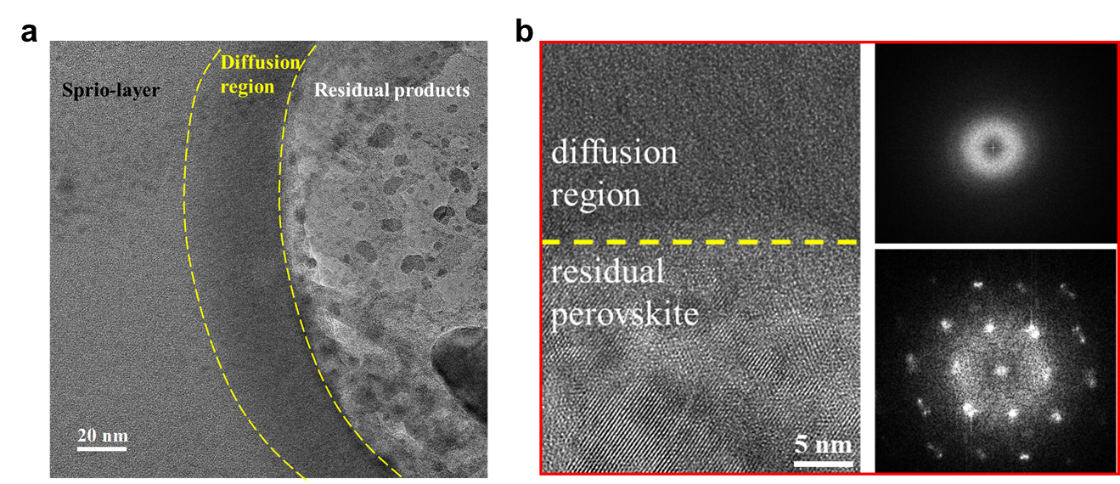


**Figure S7. TEM image.** The HRTEM image of diffusion-region in Spiro-OMeTAD layer at the interface of Spiro-OMeTAD/perovskite.


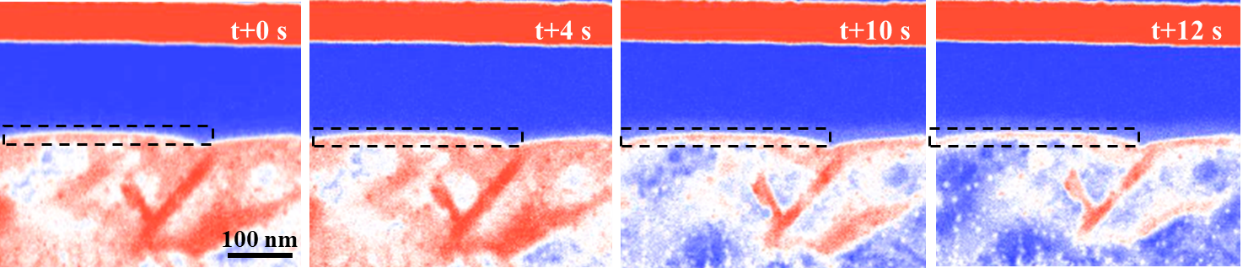


**Figure S8. ADF-STEM images.** Time-lapsed ADF-STEM images of variations of perovskite/HTL interface derived from iodide diffusion dynamics.


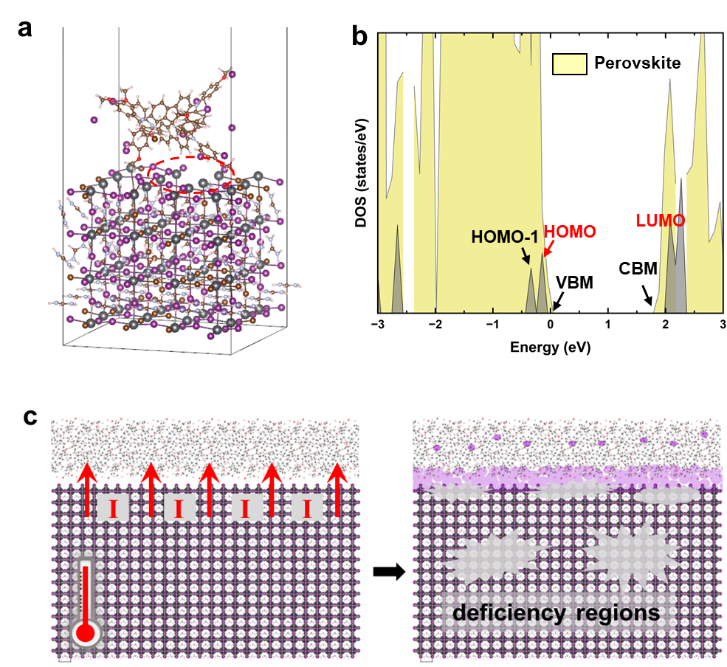


**Figure S9. DFT calculations. a)** Side view of atomic models of perovskite slabs and Spiro-OMeTAD. **b)** Density of states of the iodide ions diffused Spiro-OMeTAD molecule on perovskite surface, iodide ion concentration is 8%. **c)** Schematically illustration of iodide diffusion in perovskite.


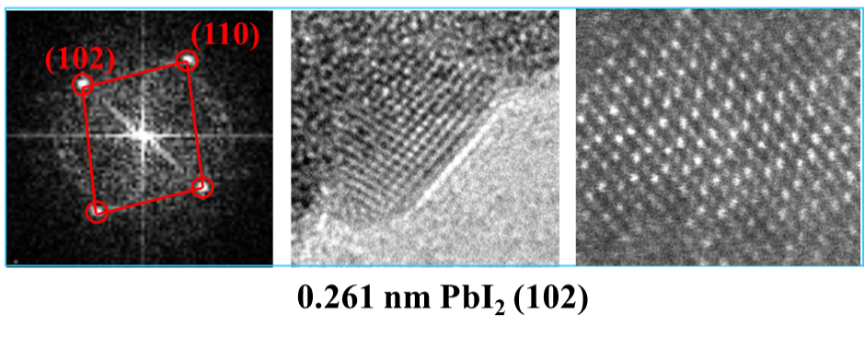


**Figure S10. Atomic-scale analysis of edge particles.** HRTEM, atomic-resolved STEM image and corresponding FFT image indicating the PbI_2_ crystal.


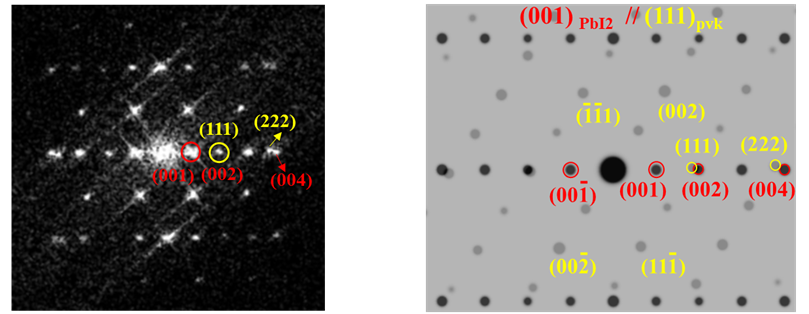


**Figure S11. FFT images.** The experimental and simulated FFT images of PbI_2_/perovskite topo-coherent structure.


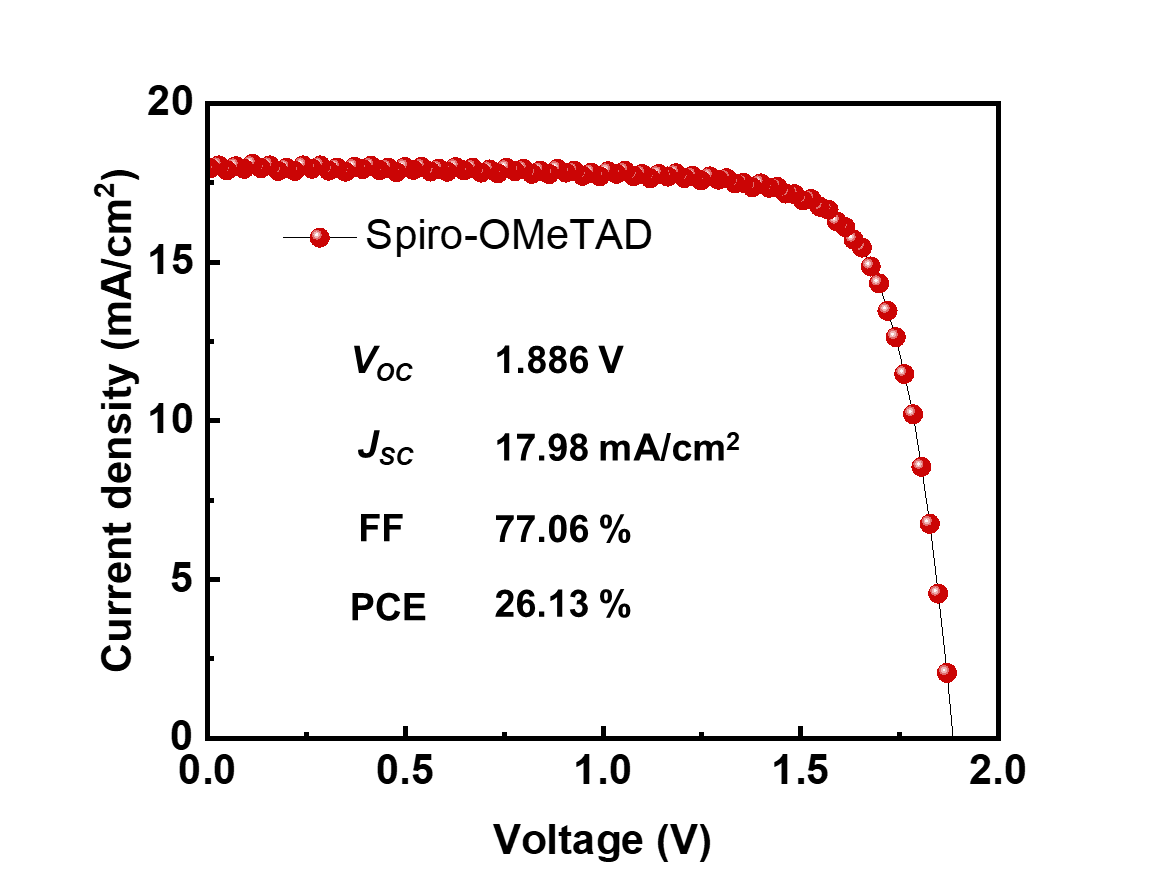


**Figure S12. *J-V* curve.** The J-V curve and performance parameters of Spiro-OMeTAD based tandem device.


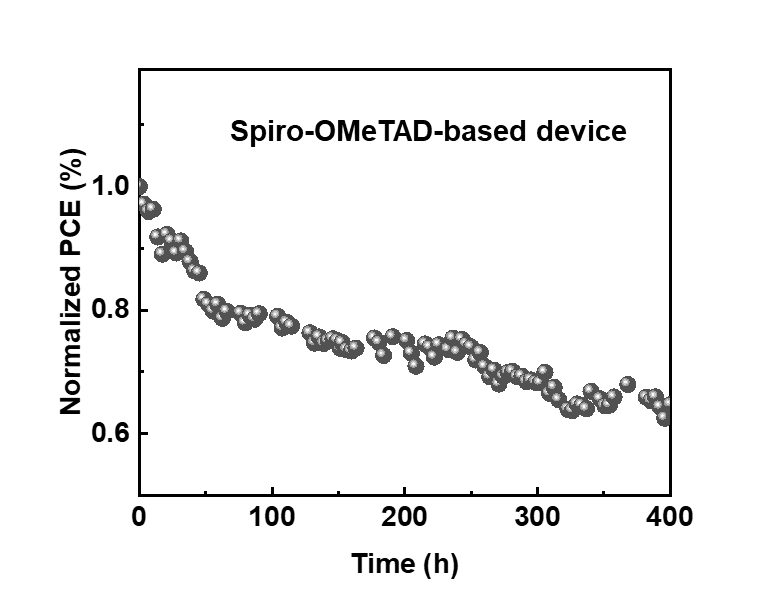


**Figure S13.** **Thermal stability tests.** MPP tracking of Spiro-OMeTAD based devices.
